# Leptomeningeal enhancement in multiple sclerosis: a focus on patients treated with hematopoietic stem cell transplantation

**DOI:** 10.3389/fneur.2024.1373385

**Published:** 2024-06-05

**Authors:** Leonardo Marchi, Alice Mariottini, Vittorio Viti, Andrea Bianchi, Chiara Nozzoli, Anna Maria Repice, Riccardo Boncompagni, Andrea Ginestroni, Valentina Damato, Alessandro Barilaro, Stefano Chiti, Riccardo Saccardi, Enrico Fainardi, Luca Massacesi

**Affiliations:** ^1^Department of Neurosciences, Drug and Child Health, University of Florence, Florence, Italy; ^2^Department of Neurology 2 and Tuscan Region Multiple Sclerosis Referral Center, Careggi University Hospital, Florence, Italy; ^3^Neuroradiology Unit, Careggi University Hospital, Florence, Italy; ^4^Cell Therapy and Transfusion Medicine Unit, Careggi University Hospital, Florence, Italy; ^5^Health Physics Unit, Careggi University Hospital, Florence, Italy; ^6^Department of Experimental and Clinical Biomedical Sciences, University of Florence, Florence, Italy

**Keywords:** multiple sclerosis, leptomeningeal enhancement, magnetic resonance imaging, autologous hematopoietic stem cell transplantation, transplant, chronic inflammation, biomarker

## Abstract

**Background:**

Leptomeningeal enhancement (LME) is considered an MRI marker of leptomeningeal inflammation in inflammatory neurological disorders, including multiple sclerosis (MS). To our knowledge, no disease-modifying therapies (DMTs) have been demonstrated to affect LME number or morphology so far.

**Methods:**

Monocentric study investigating the frequency and number of LME in a cohort of people with (pw)MS who performed a 3 T brain MRI with a standardized protocol (including a post-contrast FLAIR sequence), and exploring the impact of autologous hematopoietic stem cell transplantation (AHSCT) on this marker. In a longitudinal pilot study, consecutive MRIs were also analyzed in a subgroup of pwMS, including patients evaluated both pre- and post-AHSCT.

**Results:**

Fifty-five pwMS were included: 24/55 (44%) had received AHSCT (AHSCT group) and 31 other treatments (CTRL group). At least one LME was identified in 19/55 (35%) cases (42 and 29% in the AHSCT and CTRL groups, respectively; *p* = 0.405). In the AHSCT group, LME number correlated with age at AHSCT (*R* = 0.50; *p* = 0.014), but not with age at post-treatment MRI. In the longitudinal pilot study (*n* = 8), one LME disappeared following AHSCT in 1/4 patients, whereas LME number was unchanged in the remaining four pwMS from the CTRL group.

**Discussion:**

These results suggest that AHSCT may affect development and persistence of LME, strengthening the indication for early use of effective therapies bioavailable within the central nervous system (CNS), and therefore potentially targeting compartmentalized inflammation.

## 1 Introduction

Multiple sclerosis (MS) is a chronic inflammatory disease of the Central Nervous System (CNS), characterized by demyelinating lesions and axonal damage (1). The presence of inflammatory cell infiltrates in the leptomeninges of MS patients has been described for several years in histopathological studies (2–5). Leptomeningeal inflammation ranges from sparse infiltrates of inflammatory cells to well-organized structures that resemble lymphatic tissue, the latter defined as Ectopic Lymphoid Follicle-like structures (ELFs) (6). It has been hypothesized that ELFs correspond to chronic inflammation compartmentalized within the CNS and contribute to disease progression through the release of soluble factors (i.e., cytokines and chemokines) that promote cortical damage (7, 8).

ELFs can be visualized with Magnetic Resonance Imaging (MRI) in T2/fluid-attenuated inversion recovery (FLAIR) sequences performed after the injection of gadolinium-based contrast agent, possibly due to increased permeability of the blood–brain barrier (BBB) within ELFs. These radiological findings are defined as Leptomeningeal Enhancement (LME) (9). However, LME is not a finding specific to MS, as it was observed with similar frequency in MS patients and patients with other inflammatory and non-inflammatory diseases of the CNS (10).

In MS patients, LME is reported with a variable frequency across different studies, being the proportion higher when adopting high-magnetic field (7-Tesla—7 T) MRI compared to a 3 T magnet. Typically, the frequency of LME is higher in the progressive than in the relapsing–remitting (RR) form of the disease (9, 11). A direct association between LME and age, disease duration, and Expanded Disability Status Scale (EDSS) score was reported in the literature, but with some inconsistencies across studies. Patients with LME had a lower total brain and cortical volume (9, 12, 13), but the spatial association between LME and cortical lesions detected by MRI remains controversial (14, 15). LME tends to remain stable over time. Rare cases of disappearance of LME following high-dose steroids were reported (16), but no disease-modifying therapies (DMTs) have been demonstrated to affect LME number or morphology so far (17–19).

To our knowledge, there are no data on the prevalence of LME in patients with MS treated with autologous hematopoietic stem cell transplantation (AHSCT), a hematological procedure endorsed as a standard of care for the treatment of aggressive RR-MS refractory to conventional DMTs (20).

## 2 Materials and methods

### 2.1 Aim of the study

The main aim of the study was to describe the prevalence and characteristics of LME in MS patients treated with AHSCT compared to MS patients treated with DMTs/untreated, exploring differential correlations between LME number and clinical-demographic characteristics in the two groups.

The impact of AHSCT on LME number was then explored in a pilot series of patients evaluated both before and after AHSCT at pre-defined timepoints, and in control patients not receiving AHSCT who had longitudinal MRI follow-up.

### 2.2 Patient population

Consecutive patients affected by MS in clinical follow-up at the Neurology 2 Department of the Careggi University Hospital in Florence, Italy, who had performed at least one 3 T brain MRI with a standardized protocol (including post-contrast volumetric FLAIR) at the Neuroradiology Unit of the same hospital over an 18-month period.

The MS cohort included RR- and secondary progressive (SP-) MS patients diagnosed according to the Poser and McDonald criteria (21, 22). Patients were further stratified according to the previous treatment with AHSCT into the AHSCT group and control (CTRL) group, the latter including those cases who had not undergone AHSCT. MS patients not treated with AHSCT who are in clinical follow-up at our center and who performed a brain MRI at our facility over the pre-defined period were consecutively included in the CTRL group.

AHSCT patients received the transplant in an open-label monocentric study in collaboration with the Cell Therapy and Transfusion Medicine Unit of the Careggi University Hospital, according to the inclusion/exclusion criteria of the center, as previously reported (23). Briefly, RR- or SP-MS patients were eligible for transplant if they showed highly active disease or disability progression with signs of inflammation despite receiving treatment with high efficacy DMTs approved for MS; PP-MS and patients with medical conditions contraindicating the procedure were excluded. Patients were mobilized with cyclophosphamide 4 g/m^2^ body surface area and granulocyte-colony stimulating factor (G-CSF; 10 μg/kg per day); the conditioning protocol used was the intermediate intensity regimen BEAM+ATG (20), encompassing BCNU (Carmustine) 300 mg/m^2^ at on day −6, ARA-C (Cytosine-Arabinoside) 200 mg/m^2^/day and VP-16 (Etoposide) 200 mg/m^2^/day from day −5 to day −2, and Melphalan 140 mg/m^2^ at on day −1; rabbit anti-thymocyte globulin (ATG, Thymoglobulin™, Sanofi) was added at a dose of 3.75 mg/kg/die day on day +1 and + 2 (total dose 7.5 mg/Kg).

### 2.3 MRI analysis

All the patients underwent a brain MRI with the same scanner and standardized protocol. Two MRI examinations taken at least 6 months apart were available for a subset of patients, including a pre-transplant MRI scan and at least one MRI performed after AHSCT for those in the AHSCT group.

Patients treated with AHSCT underwent brain MRIs at pre-defined timepoints, i.e., before AHSCT, at month 6 and 12 after AHSCT and then yearly; no unscheduled MRIs were analyzed in this study. The time interval between AHSCT and post-AHSCT MRI was dependent on the time interval between AHSCT and 3 T machine purchase.

The scans were obtained with a 3 T MRI scanner (Ingenia, Philips Healthcare, The Netherlands). The standardized protocol included a 3D-volumetric T2-FLAIR sequence acquired 3–4 min after the injection of gadolinium-based (0.1 mL/kg) contrast, and 3D T1 post-contrast sequence. A T2-FLAIR sequence acquired before gadolinium administration was not available as it was not included in the protocol of the Center.

LME was defined according to the literature as “high signal intensity within the subarachnoid space that is substantially greater than that of brain parenchyma” (9). The presence of LME foci was investigated by analyzing all the slices of T2-FLAIR scans for each patient; the analyses were performed by two raters trained in neuroimaging, who were blind to the demographical and clinical information of the patients. Cohen’s kappa (κ) for inter-rater agreement was 0.8. Longitudinal changes in LME number were confirmed by a third trained rater.

Contrast enhancement in the context of the pachymeninges (Dura Mater Enhancement, DME) and around the meningeal blood vessels (meningeal Vessel Wall Enhancement, VWE) was also evaluated (24).

### 2.4 Statistical methods

Baseline characteristics of the study population are reported as median (range) or number (frequency) for continuous and dichotomous variables, respectively. The Mann–Whitney U test or Chi-square were used to compare the baseline characteristics between the groups, as appropriate according to data distribution. Bivariate Spearman correlation was used to explore correlations between the number of LME and baseline characteristics of the patients, corrected for age at MRI. The statistical analysis was performed using the Statistical Package for the Social Sciences software (SPSS, IBM, Armonk, NY, United States, version 25.0).

## 3 Results

### 3.1 Characteristics of the patient population

**Figure 1:**
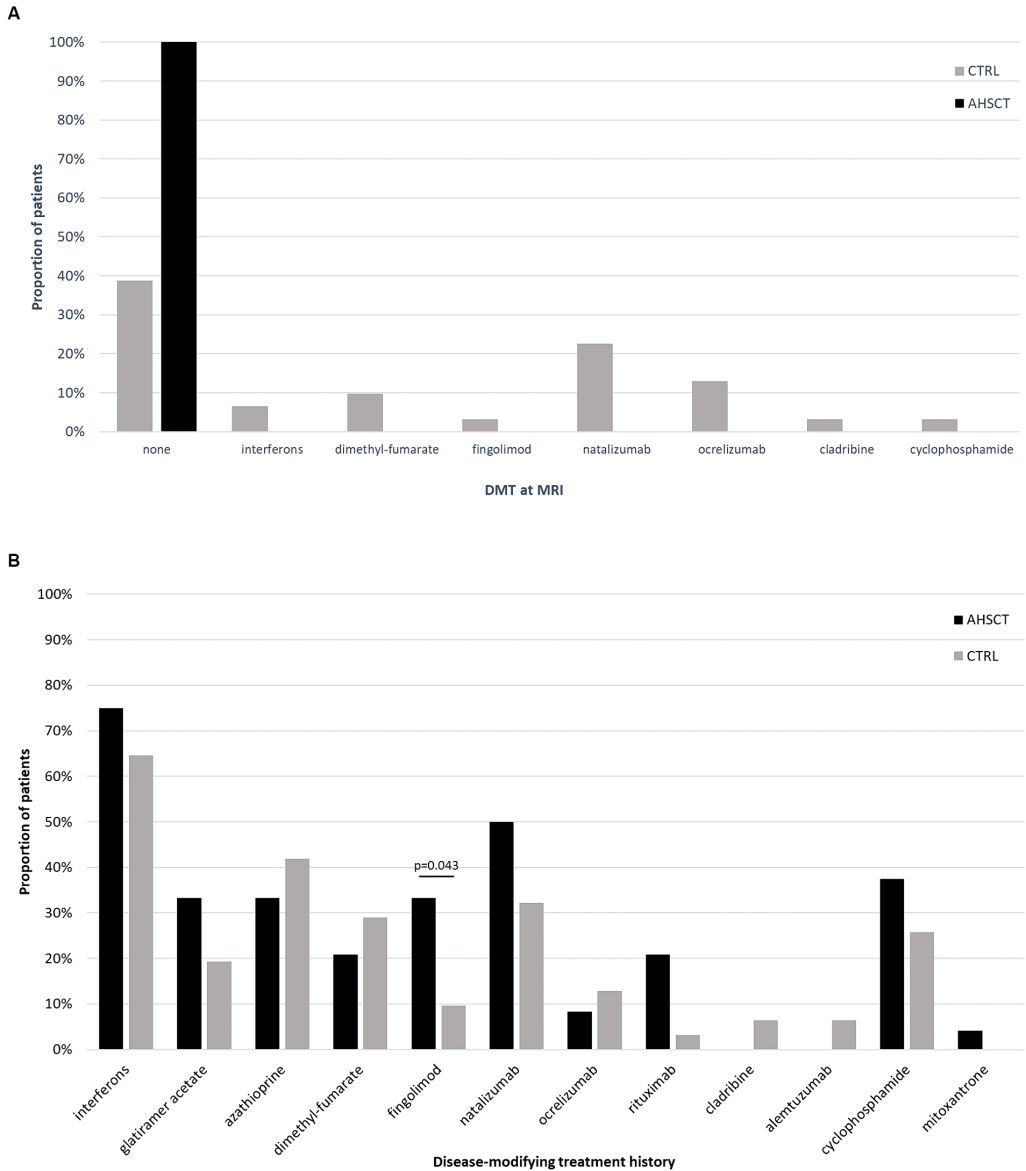
Proportion of patients from the AHSCT and CTRL groups who were receiving active treatment with each DMT at the time of MRI **(A)** or who had received each DMT since MS diagnosis **(B)**. All the patients from the AHSCT group were untreated at the time of MRI, whereas 61% of patients in the CTRL group were receiving treatment with DMTs. No significant differences were observed in treatment history between the two groups, except for a higher proportion of patients from the AHSCT group who had received fingolimod.

Fifty-five MS patients (40 RR-MS; 15 SP-MS) were included (Table 1). Twenty-four/55 (44%) patients had received AHSCT a median of 44 (5–229) months before the MRI scan. All the patients in the AHSCT group were free from DMTs at the time of MRI; 61% of the cases in the MS CTRL group were receiving active treatment (Figure 1A). DMTs received prior to MRI are detailed in Figure 1B. No differences in proportion of patients treated with each DMT were observed between AHSCT and CTRL patients, except for fingolimod, which was received by 33 and 10% of cases from the AHSCT and CTRL groups, respectively (*p* = 0.043). Most of the patients were affected by RR-MS (Table 1). No differences were observed between the two groups in clinical-demographic characteristics, except for the duration of treatment with DMTs and the number of previous DMTs, that were both higher in the AHSCT group than in the CTRL group (*p* = 0.024 and < 0.001, respectively; Table 1).

**Table 1:** Clinical-demographic characteristics of the overall cohort and the AHSCT and CTRL groups at the time of the MRI scan.

	Overall (*n* = 55)		AHSCT group (*n* = 24)		CTRL group (*n* = 31)		
	Median	(Range)	Median	(Range)	Median	(Range)	*p* Value
Age, years	46	(22–74)	46	(29–57)	43	(22–74)	0. 753
Disease duration, years	16	(0–52)	17	(6–31)	13	(0–52)	0.391
Progressive phase duration, years	6	(1–24)	6	(1–24)	6	(1–8)	0.620
Treatment duration, years	11	(0–26)	13	(5–26)	10	(0–25)	0.024
EDSS	4.0	(0.0–7.0)	3.5	(1–7)	4.0	(0.0–7.0)	0.385
Number of previous DMTs	3	(0–7)	4	(2–7)	2	(0–5)	<0.001
	*n*	(%)	*n*	(%)	*n*	(%)	*p* value
Gender, female	39	(71%)	20	(83%)	19	(61%)	0.133
MS phenotype: RR-MS	40	(73%)	16	(67%)	24	(77%)	0.375
Patients receiving treatment with DMTs at MRI	19	(35%)	0	(0%)	19	(61%)	<0.001

AHSCT, Autologous hematopoietic stem cell transplantation; CTRL, Controls, i.e., patients who did not receive AHSCT; DMTs, Disease-modifying treatments; EDSS, Expanded disability status scale; MRI, Magnetic resonance imaging; MS, Multiple sclerosis; RR-, Relapsing–remitting.

### 3.2 Leptomeningeal enhancement

**Figure 2:**
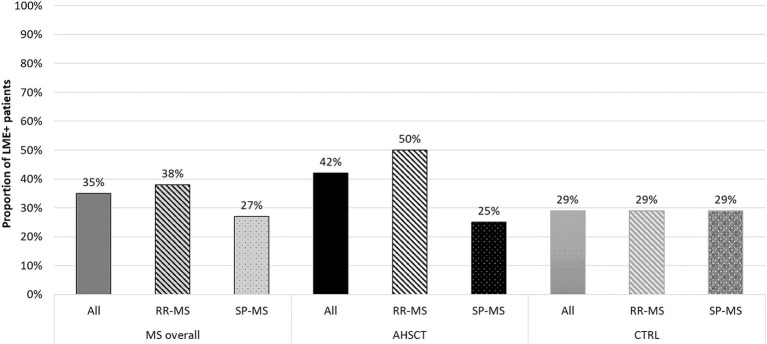
Proportion of LME-positive patients in the overall MS cohort and in the AHSCT and CTRL groups, and in patients from each group stratified according to the MS form (relapsing–remitting, RR, or secondary-progressive, SP). RR-MS patients from the AHSCT group tended to higher proportion of LME positivity compared to those in the CTRL group.

At least one LME focus was identified in 19/55 (35%) MS patients, including 15/40 (38%) RR-MS and 4/15 (27%) SP-MS (*p* = 0.537; Figure 2).

Ten/24 (42%) cases from the AHSCT group and 9/31 (29%) from the CTRL group showed at least one LME. Fifty percent of the RR-MS patients from the AHSCT group were LME-positive versus 29% of those in the CTRL group (Figure 2).

**Figure 3:**
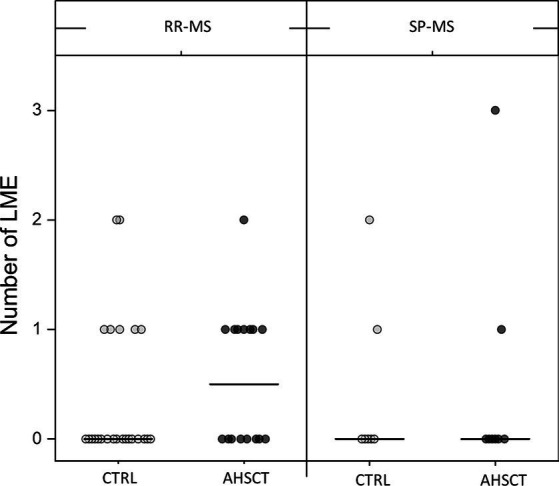
Number of LME in the AHSCT and CTRL groups, stratified according to the MS form (relapsing–remitting, RR, or secondary-progressive, SP). Median values are represented by a line.

LME-positive patients showed a median of 1 (range 1–3) LME foci, ranging between 1 and 2 and 1 and 3 in the CTRL and AHSCT groups, respectively (Figure 3).

### 3.3 Factors associated with leptomeningeal enhancement

In the AHSCT group, the number of LME was directly correlated with age at AHSCT (*R* = 0.50; *p* = 0.014), but not with age at the time of MRI, and it was inversely correlated with the time between AHSCT and MRI (*R* = −0.49; *p* = 0.018) (Table 2). MS duration showed a mild negative correlation with LME number in the whole cohort. No other significant correlations between LME number and clinical-demographic characteristics were observed in the overall cohort or CTRL group.

**Table 2:** Correlations between LME number and clinical-demographic characteristics of MS patients at the time of the MRI scan, corrected for age at the MRI.

	MS overall (*n* = 55)		AHSCT (*n* = 24)		CTRLS (*n* = 31)	
	*R*	(*p* value)	*R*	(*p* value)	*R*	(*p* value)
Age, years	0.07	(0.590)	0.13	(0.540)	0.07	(0.727)
Disease duration, years	−0.27	(0.044)	−0.40	(0.060)	−0.17	(0.379)
Treatment duration, years	−0.10	(0.92)	−0.41*	(0.049)	0.10	(0.615)
Progressive phase duration, years	−0.29	(0.341)	−0.57	(0.234)	0.11	(0.837)
*N* prior DMTs	0.08	(0.542)	−0.08	(0.705)	0.13	(0.477)
EDSS	−0.12	(0.929)	−0.06	(0.787)	0.02	(0.900)
Age at AHSCT, years	N.A.	N.A.	0.50*	(0.014)	N.A.	N.A.
Time AHSCT-MRI, years	N.A.	N.A.	−0.49*	(0.018)	N.A.	N.A.

Significant correlations are marked with ^*^.

AHSCT, Autologous hematopoietic stem cell transplantation; CTRL, Control group; DMTs: Disease-modifying treatments; EDSS, Expanded disability status scale; LME, Leptomeningeal enhancement; MRI, Magnetic resonance imaging; MS, Multiple sclerosis; N.A., Not applicable.

### 3.4 Dural and vessel wall enhancement

Contrast enhancement was also observed in the context of the dura mater (DME) and meningeal vessels (VWE). The median DE and VWE number were 0 (0–1) and 0 (0–4), respectively. Median VWE number was 0 (0–4) and 1 (0–4) in the AHSCT and CTRL groups, respectively.

### 3.5 Longitudinal pilot study

Seriate MRI scans were available for four MS CTRL (four RR-MS) and four MS AHSCT (two RR-MS; two SP-MS). In the former group, two MRI were analyzed for each patient, taken a median of 11 (6–13) months apart. In the AHSCT group, one MRI was taken before undergoing AHSCT (baseline scan) and at least one after transplant according to a pre-defined schedule, with a median interval of 16 (7–27) months between the pre-AHSCT scan and the latest follow-up available.

**Figure 4:**
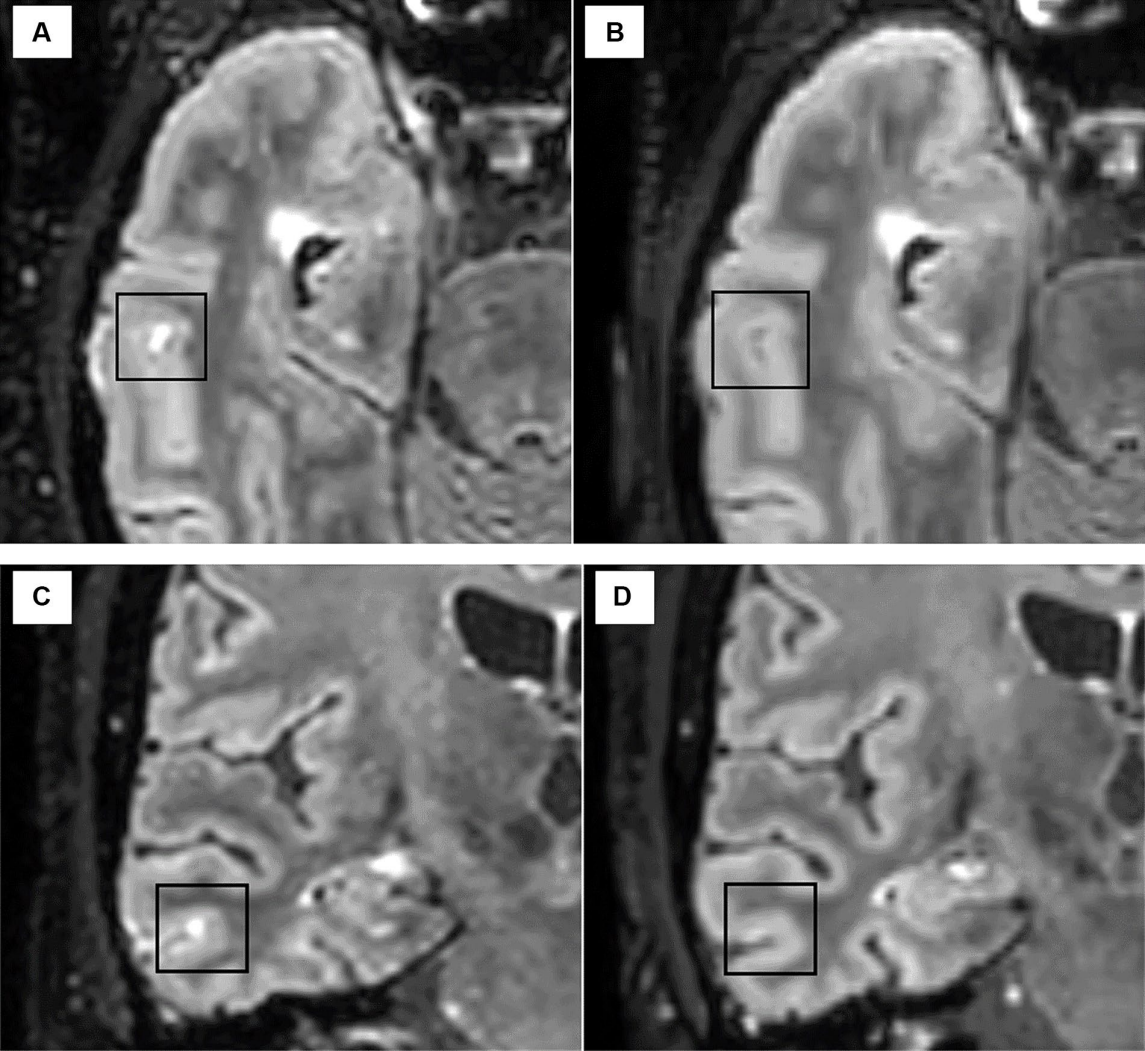
The disappearance of one LME focus was observed in one patient after AHSCT from the pre-treatment scan [axial **(A)** and coronal **(C)**] to the MRI scan taken at 6 months after AHSCT [axial **(B)** and coronal **(D)**].

In AHSCT patients, the disappearance of one LME detected in the baseline scan was observed in one/four cases at month 6 after AHSCT (Figure 4), and the number of LMEs remained stable at a subsequent exam taken 6 months later. The number and morphology of LME were unchanged at follow-up MRIs (6 and 12 months after AHSCT) in the remaining three patients.

No changes in the number of LME (median 1, range 1–2) were observed in the four patients from the MS CTRL group in the follow-up MRI scan.

## 4 Discussion

In the present study, the presence of LME was explored in people with MS, as it has been proposed as a potential MRI biomarker of compartmentalization of the immune response in the CNS. We observed a prevalence of LME similar between RR-MS and SP-MS patients, a finding conflicting with most of the published studies (9, 24) that show a significantly higher frequency of LME in progressive MS compared to RR-MS. Such difference could be attributed, at least in part, to a selection bias toward patients affected by highly active or aggressive MS recruited in the AHSCT program, who could bear signs of compartmentalized inflammation early on during disease course. A trend for a higher frequency of LME-positive cases was indeed observed in RR-MS patients treated with AHSCT compared to RR-MS patients from the CTRL group.

It has been hypothesized that some of the currently available DMTs may act on leptomeningeal follicles, however longitudinal studies with rituximab, dimethyl-fumarate, and teriflunomide (17–19) did not show any reduction in LME number over time; on the contrary, an increase in the number of LME positive patients and LME foci was observed in dimethyl-fumarate and teriflunomide treated MS patients over a 24-month follow-up (17). To our knowledge, only anecdotal cases of LME disappearance following high-dose IV steroid therapy have been described so far (16), which could indeed be due to a transient modification of the BBB permeability induced by the treatment. The sample size and heterogeneity of treatment history did not allow us to explore the effect of DMTs on LME number. However, the two groups did not differ in terms of prior use of DMTs, except for fingolimod and, to our knowledge, no data are available supporting the effect of DMTs on LME formation/persistence.

In this respect, the bioavailability of a drug within the CNS would be a pre-requisite for acting on compartmentalized inflammation and, likely, for affecting LME persistence. The chemotherapy drugs administered during the conditioning regimen in the BEAM protocol are able to cross the BBB, with the highest penetration rate for carmustine (25). BEAM-based AHSCT may therefore plausibly affect compartmentalized inflammation thanks to the use of chemotherapy drugs crossing the BBB. In this study, the prevalence of LME was not significantly different between patients who had received AHSCT and those who had not. However, we observed a direct correlation between the number of LME and age at AHSCT, but not with age at MRI. Interestingly, the number of LME was inversely correlated with the time between AHSCT and the (post-AHSCT) MRI. Although still speculative, as a direct correlation between age and LME frequency is described in the literature (although not consistently across studies) (10, 12, 13, 24), such findings could suggest that AHSCT may halt the formation of new ELFs. If this hypothesis was true, it could strengthen the indication for early treatment with high efficacy DMTs active within the CNS, in order to prevent further accumulation of ELFs and possibly related disability progression. However, we did not detect any correlation between the number of LME and age at MRI in the CTRL group. As such correlation is reported in wide cohorts (12, 24), but not consistently described in small patient populations like that included in this study (13), the detection of a potential weak correlation between LME number and age at MRI could be undermined by the small sample size of the CTRL group.

In the longitudinal pilot study, a reduction in LME number was observed following AHSCT in one out of four cases analyzed, a finding that needs to be confirmed in larger patient populations. In the remaining three patients, the number and morphology of LMEs were not affected by AHSCT, although the observation was performed over a short-term follow-up. No changes in LME number were observed in the four CTRL patients evaluated longitudinally. However, in our opinion, it cannot be excluded that irreversible structural modification had already occurred in the BBB and structures within ELFs and that other elements may be responsible for their persistent enhancement. In this latter hypothesis, if inflammatory infiltrates within ELFs were effectively removed by a DMT, LME could persist indefinitely over time without actually corresponding to ELFs. If this was true, a possible dissociation between this marker and clinical outcomes may be observed over long-term. However, the small sample size and the lack of a long-term prospective follow-up after AHSCT did not allow us to further explore this intriguing hypothesis.

Our study has several limitations. First of all, the sample size in the longitudinal pilot study was small and the post-AHSCT follow-up relatively short, therefore these findings should be considered as exploratory. Comparisons between the AHSCT and CTRL groups should be taken with caution for a possible selection bias in the CTRL group, as no matching for clinical-demographic characteristics was performed. Serum biomarkers could not be analyzed as biological samples were not available due to the retrospective design of the study. Although the frequency and number of DE and VWE were recorded, a formal analysis of possible correlations with clinical-demographic variables was not performed as their role in MS is debated, and their detection and interpretation were challenging due to potential artifacts.

## 5 Conclusion

In the present study, a similar prevalence of LME was observed between MS patients who had received AHSCT and those who had not. However, the observation of a direct correlation between the number of LMEs and patients’ age at AHSCT, but not at MRI, and the disappearance of one LME focus after AHSCT in one case suggest that AHSCT may halt the formation of new LMEs. If this hypothesis was true, early treatment with high-efficacy therapies reaching the CNS compartment could reduce leptomeningeal inflammatory infiltrates organized in ELFs and possibly prevent (or revert) the compartmentalization of inflammation. Longitudinal prospective studies with long-term follow-up are needed to clarify if LME can represent a marker of response to treatments bioavailable within the CNS.

## Data availability statement

The datasets presented in this article are not readily available according to privacy regulations. Aggregated data will be shared upon motivated request to the corresponding author. Requests to access the datasets should be directed to alice.mariottini@unifi.it.

## Ethics statement

The studies involving humans were approved by Comitato Etico Area Vasta Centro—Regione Toscana. The studies were conducted in accordance with the local legislation and institutional requirements. The participants provided their written informed consent to participate in this study.

## Author contributions

LeM: Writing – original draft, Investigation. AM: Writing – review & editing, Writing – original draft, Visualization, Methodology, Investigation, Formal analysis, Conceptualization. VV: Writing – original draft, Resources, Investigation, Data curation. ABi: Writing – review & editing, Resources. CN: Writing – review & editing, Resources. AR: Writing – review & editing, Resources. RB: Writing – review & editing, Resources. AG: Writing – review & editing, Resources. VD: Writing – review & editing, Resources. ABa: Writing – review & editing, Resources. SC: Writing – review & editing, Resources. RS: Writing – review & editing, Resources. EF: Writing – review & editing, Investigation. LuM: Writing – review & editing.
